# Mindfulness-based eating awareness training (MB-EAT) decreases emotional eating behavior among Hong Kong university students: a randomized controlled trial

**DOI:** 10.1007/s10865-026-00673-3

**Published:** 2026-05-24

**Authors:** Dalinda Isabel Sanchez Vidaña, Daniel Kwasi Ahorsu, Lynette McCormack, Roy Rongyue Zeng, Tiev Miller, Nestor Vinas Guasch, Ngai-Man Jackie Chan, Kenneth Ka-Hei Lo, Pablo Cruz Gonzalez, Benson Wui-Man Lau, Way Kwok-Wai Lau

**Affiliations:** 1 Department of Rehabilitation Sciences, The Hong Kong Polytechnic University, Hong Kong, China; 2 Mindfulness Training Centre, Hong Kong, China; 3 Department of Special Education and Counselling, The Education University of Hong Kong, Hong Kong, Hong Kong SAR China; 4 Independent Researcher, Attentive Weight Management, Rockhampton, Australia; 5 Department of Food Science and Nutrition, The Hong Kong Polytechnic University, Hong Kong, China; 6 Rehabilitation Research Institute of Singapore, Nanyang Technological University, Singapore, Singapore; 7 School of Nursing and Health Sciences, The Hong Kong Metropolitan University, Hong Kong, Hong Kong SAR China

**Keywords:** Eating behavior, Emotional eating, Mental health, Mindfulness, Overeating, University students

## Abstract

University students in Hong Kong facing academic stress are prone to engage in emotional eating, that has detrimental physical and psychological effects. Addressing emotional eating through interventions like Mindfulness-Based Eating Awareness Training (MB-EAT), which effectively treats clinical eating disorders, is crucial, yet its effectiveness among students remains underexplored. A parallel group randomized controlled trial with 46 students (42 female, 4 male) evaluated the effect of a 10-week MB-EAT intervention, delivered through Zoom, on emotional eating (primary outcome), measured by the Salzburg Emotional Eating Scale at pre-, mid-, and post-intervention, with secondary measures of mood and mindfulness evaluated pre- and post-intervention. Students in the MB-EAT group showed greater reductions in emotional eating and improvements in depression and mindful eating. MB-EAT may offer greater long-term benefits for students with emotional eating by reducing negative psychosocial effects, helping students better cope with stress through emotion regulation and mindful eating skills.

## Introduction

Emotional eating refers to the tendency to overeat in response to both negative (e.g., anxiety or irritability) and positive emotions (e.g., happiness), as well as stress (Cardi et al., 2015; Debeuf et al., 2020; Evers et al., 2013; Lattimore, 2020). In emotional eating, individuals often seek to manage strong emotions or stress by eating, even when they are not physically hungry (Debeuf et al., 2020). This behavior can lead to overeating and an increase in caloric intake (Watford et al., 2019). People exposed to high levels of stress are more prone to develop maladaptive behaviors, such as emotional eating, as a coping mechanism for emotional distress (Sze et al., 2021).

University students experience significant levels of stress triggered by high academic expectations, financial concerns, sleep problems, and high expectations from peers and family to excel (Ling & Zahry, 2021; Pedrelli et al., 2015). High levels of stress in this population can trigger emotional eating that has been associated with lower sleep quality, binge eating, psychological problems like depression, and higher stress sensitivity (Brown & Ryan, 2003; Brown et al., 2007; Cardi et al., 2015). The increased calorie intake caused by emotional eating behavior can lead to overweight and obesity (Burnatowska et al., 2023; Dakanalis et al., 2023), factors that are associated with a higher risk of developing chronic medical conditions, including diabetes, hypertension, and some types of cancer, including colorectal cancer (Abdullah et al., 2010; Riaz et al., 2018; Ungvari et al., 2024). Therefore, it is crucial to develop effective strategies to regulate eating behavior in response to emotions and stress to promote students’ well-being.

Emotional eating has been identified as a major health concern among university students, with a prevalence ranging from 15% to 47.5% (Cheng & Wong, 2021; Grajek et al., 2022; Muharrani et al., 2018). In China, the prevalence of emotional eating is estimated to range between 14.5% and 52.7% (He et al., 2020; Liu et al., 2020). Comparably, more than 14% of female students in Hong Kong have reported emotional eating in response to negative emotions (Sze et al., 2021). Particularly, stress as one of the psychosocial factors positively associated with emotional eating is also prevalent among university students in Hong Kong (Du et al., 2022; Ling & Zahry, 2021; Sze et al., 2021). 22.2% of them may suffer from medium or higher levels of stress (Shek et al., 2022). Similar findings were reported in another study showing higher levels of stress in students aged 18–29 years (Kwok & Ng, 2016). The COVID-19 pandemic further exacerbated the problem in young adults aged between 18 and 25 years, reporting higher stress levels compared to other age groups. Interestingly, this trend was partially attributed to academic-related concerns (e.g., academic progress and learning support) (Fong et al., 2020). Taken altogether, it is clear that university students are likely to benefit from interventions designed to reduce both stress and emotional eating to mitigate the negative impact on their physical and psychological well-being and academic performance.

Treatments for emotional eating include cognitive behavioral therapy, acceptance and commitment therapy, behavioral weight loss, intensive counseling, dialectical behavioral therapy, and mindfulness-based interventions (Smith et al., 2023). These interventions mainly focus on recognizing and understanding emotional eating, recognizing stressors and resulting emotions, and the development of emotion regulation skills (Smith et al., 2023). In a recent meta-analysis, pure mindfulness-based interventions showed a higher effect size for emotional eating compared to other interventions or a combination of interventions (Smith et al., 2023). These findings suggest that mindfulness-based interventions are a promising therapeutic option to improve emotion regulation skills and reduce emotional eating behavior. The awareness and negative emotion can reciprocally weaken each other; the negative emotion will be accumulated in the absence of awareness (Zhou et al., 2020).

Mindfulness is described as the awareness that emerges through intentionally paying attention in the present moment non-judgmentally (Kabat-Zinn, 2003), i.e., without conceptual or emotional classification (Brown et al., 2007). Mindfulness interrupts habitual patterns of thought, emotion, and behavior to allow for a more adaptive response to occur (Brown et al., 2007; Kabat-Zinn, 2003). Mindfulness is conceptualized as a state that is attained and then practiced in mindfulness meditation (Lau et al., 2006) and as a trait, described as trait or dispositional mindfulness, that refers to one’s predisposition to be mindful in daily life (Baer et al., 2006). Mindfulness practices can cultivate greater state mindfulness, which, over time, contributes to increased trait mindfulness (Kiken et al., 2015; Tang et al., 2016). The relationship between mindfulness and healthy eating behavior, such as reduced emotional eating, has been previously established (Katterman et al., 2014; Levin et al., 2014; Macht, 2022; Watford et al., 2019).

Mindfulness-Based Eating Awareness Training (MB-EAT) is an intervention originally designed for binge eating (Kristeller et al., 2014). It has been reported to promote awareness of bodily experiences related to appetite signals of physical hunger; sensory perception of satiety, and taste satisfaction; emotional triggers of overeating (Kristeller & Hallett, 1999), cue reactivity, impulse control, and stress (Lattimore, 2020). Evidence from clinical studies evaluating the effect of MB-EAT has shown improved emotional impulse regulation and mood, suggesting improved self-regulation of eating patterns (Kristeller & Wolever, 2014; Kristeller et al., 2014; Lattimore, 2020). Therefore, this study aimed to evaluate the effect of a 10-week MB-EAT program on the emotional eating behaviors of university students classified with emotional eating. To identify potential factors that may contribute to treating emotional eating, correlation analysis was conducted. We hypothesized that university students with emotional eating who receive a 10-week MB-EAT program would report a significantly greater reduction in emotional eating behavior when compared with students classified with emotional eating in the waitlist group. To our knowledge, this is the first randomized controlled trial to evaluate the efficacy of MB-EAT on emotional eating in university students in Hong Kong.

## Materials and methods

### Study design

This study was a parallel group randomized controlled trial to explore the effects of a 10-week MB-EAT intervention on emotional eating in university students in Hong Kong. The study protocol was registered at the Hong Kong University Clinical Trials Registry (HKUCTR), with the Study Identifier HKUCTR-3035.

### Ethical considerations

Ethical approval was obtained from The Hong Kong Polytechnic University (Ref. HSEARS20230709001-02). The research activities conducted complied with the Declaration of Helsinki Principles and the guidelines of The Hong Kong Polytechnic University. Informed consent was obtained from the participants before the study began. Other ethical issues, such as confidentiality and anonymity, were assured.

### Participants

University students (undergraduate, postgraduate, local, non-local) over 18 years of age who obtained a score ≥ 2.8 in the emotional eating subscale of the Dutch Eating Behavior Questionnaire (DEBQ) were recruited for this study. This threshold distinguishes the first third DEBQ scores from the lower two-thirds scores based on a sample of 1384 people and has been validated to separate emotional eaters from the healthy population (Macht, 2022). Students who were able to read and understand English were included in the study because the intervention was designed to target both international and local students from tertiary institutions where the medium of instruction is English. Therefore, both the intervention and assessments were conducted in English. Participants were excluded when meeting the following criteria: (1) they had been diagnosed with anorexia or bulimia nervosa with a metabolic condition (e.g., diabetes, insulin resistance); (2) self-reported substance abuse, pharmacological treatment for weight loss at the time of recruitment; (3) having prior experience in mindfulness meditation practice. The sample size was calculated a priori using the G Power 3.1 software version 3.1.9.6. To achieve an acceptable effect size estimate of 0.35 for a repeated measures design within-between interaction, 0.05 alpha, 0.80 Power for 2 groups, and 2 time points, 0.5 for correlation among the repeated measures, and 1 for non-sphericity correction, a minimum of 40 participants (20 per group) would be required. After considering an anticipated attrition rate of 10%, a total of 44 participants (22 per group) were needed.

### Recruitment

Participants were recruited from several universities in Hong Kong through social media, email, and posters. The message for recruitment contained information related to the nature of the intervention (e.g., mindfulness-based intervention, not a weight-loss intervention), the inclusion criteria (e.g., “Are you affected by emotional eating? That is, eating when experiencing strong emotions”, “Would you like to change your emotional relationship with food and eating? If so, try the Mindfulness-Based Eating Awareness Training course (Kristeller et al., 2014), and a QR code directing participants to the registration form with detailed information about the study and the informed consent statement.

### Randomization

University students with emotional eating behavior (i.e., those who got a score ≥ 2.8 in the emotional eating subscale of the DEBQ) were randomly allocated into the MB-EAT or the waitlist group using stratified randomization by matching age, gender, and BMI. A total of 46 participants were randomized to the MB-EAT and the waitlist group, each group with 23 participants, including 21 females and 2 males in each group.

### Procedure

All eligible participants were contacted by email to complete the questionnaires online at baseline. Participants were informed of their group allocation after completing the baseline assessment and were provided an orientation video according to their assigned group (Kristeller et al., 2014). The orientation video for the MB-EAT group included information about the intervention, schedule of the Zoom sessions, information about the materials needed for the sessions (e.g., food for mindful eating practice), important dates for completion of questionnaires, daily record of the meditation practice, and contact details of the principal investigator. Participants in the waitlist group received an orientation video with information about the important dates for completion of questionnaires, information about weekly email contact from the research team sharing articles on nutrition and exercise, and contact details of the principal investigator. The weekly intervention sessions were offered twice during the weekend and at different times of the day to offer the flexibility of attending the weekly Zoom sessions. In case a session was missed, the participant was contacted to schedule an individual session to cover the material of the missed session (Kristeller et al., 2014). During the intervention period, participants in the waitlist group received information about nutrition and exercise via email in the form of infographics (once weekly) to promote retention. Five weeks after starting the intervention, both the MB-EAT and waitlist groups completed the 20-item Salzburg Emotional Eating Scale to assess emotional eating behavior (primary outcome). After the 10-week intervention, participants in both the MB-EAT and waitlist groups were contacted by email to complete the questionnaires online. After completing the post-test assessments, participants in the waitlist group were offered the 10-week MB-EAT intervention.

### Intervention

The design of the MB-EAT program includes 10 weekly sessions via Zoom. Participants had the option to join one session per weekend, choosing one of 4 time slots (2 on Saturday and 2 on Sunday). The duration of each session varied from 1.5 to 2.5 h and included an introduction to the self-regulation model, meditation, mindful eating, body scan, and information on seven eating and food-related topics (e.g., binge eating triggers, taste satiety clues) (Kristeller & Jordan, 2018; Kristeller et al., 2014). A detailed description of the content per session and home practice is shown in Table 1. MB-EAT focuses on flexibility, making long-term changes for developing sustainable eating patterns regarding food choice, including more ‘indulgent’ foods (though in smaller quantities), and increasing enjoyment of eating (Kristeller & Jordan, 2018; Kristeller et al., 2014). In each session, conceptual content and practical exercises were delivered (Kristeller & Jordan, 2018; Kristeller et al., 2014). Except for mindfully eating raisins, adapted from the MBSR program, all eating practices were developed for MB-EAT, incorporating foods often identified as frequently over-eaten, including cheese and crackers, chocolate, corn chips, and cookies (Kristeller & Jordan, 2018). The MB-EAT intervention was delivered by a qualified instructor, certified through an international MB-EAT training institute, using a trauma-sensitive mindfulness approach, which means adapting mindfulness practices to be respectful and supportive of individuals with trauma histories by creating a safe and empowering environment (Duane et al., 2021). This approach was used to ensure that participants feel secure and comfortable during the practice, reducing the risk of re-traumatization and promoting more effective engagement in mindfulness exercises.

**Table 1:** Content of the weekly MB-EAT sessions and administration schedule

Week	Session	Foods used in session	Content	Home mindfulness practice (with and without audio tracks)
1	Introduction	- 4 raisins	Guided meditation practice (10 min) Introduction to the Self-Regulation Model (Inner Wisdom) and Outer Wisdom Exercise with raisins Basic meditation instruction (mindfulness meditation including guided practice)	Generl mindfulness meditation track (10 min)
2	Mindful eating (high fat food)	-Cheese and crackers	Guided meditation practice (10 min) Introduction to mini-meditation Mindful eating practice (cheese and crackers) Concept of mindful eating Quality over quantity Introduction to “KEEP in Balance”: This mindfulness-based self-monitoring tool is designed to encourage regular, small changes that reflect more mindful eating patterns. The “KEEP in Balance” questionnaire assesses individuals' mindful eating behaviors, emotional regulation related to eating, and physical activity patterns over the past week Closing meditation practice	General mindfulness meditation track (10 min) Mini-meditation (2 min without audio track and 6 min with audio track) before meals Eat one snack or meals per day mindfully (repeated for all sessions, with increasing number of meals/snacks to be eaten mindfully per day) Start the Quality over Quantity challenge Complete the “KEEP in balance questionnaire”
3	Hunger awareness, emotional eating and other triggers	NA	Guided meditation practice (10 min) Check-in on home meditation practice, food, and eating Physical hunger awareness Emotional eating and other triggers Hunger cues: Physiological versus emotional cues Guided hunger meditation Weighing Home practice revision	General mindfulness meditation track (10 or 20 min) Hunger awareness audio Eating triggers meditation 10 min Home practice: Eat when physically hungry Eat one snack or meal per day mindfully (repeated for all sessions, with increasing number of meal/snacks to be eaten mindfully per day)
4	Physical activity, taste satiety, and healing self-touch	- Chocolate cookies	Guided meditation practice (10 min) Check-in on home meditation practice, food, and eating Physical activity/pedometers Taste satisfaction practice Body scan and healing self-touch practice	General mindfulness meditation track (10 or 20 min) Mini-meditation (2 min without audio track and 6 min with audio track) before meals Taste satiety meditation 10 min Body scan and self-healing touch 10 min Monitor your physical activity Eat one snack or meal per day mindfully (repeated for all sessions, with increasing number of meal/snacks to be eaten mindfully per day)
5	Fullness awareness, food choices, eating and emotions	− 1–1.5L water - Cookies and chips	Guided meditation practice (10 min) Check-in on home meditation practice, food, and eating Fullness awareness and body satiety Fullness meditation Eating exercise: Mindful food choices (cookies versus chips) Eating and emotions Forgiveness meditation	General mindfulness meditation track (10 or 20 min) Fullness awareness meditation 10 min Mindful choice meditation 10 min Forgiveness meditation 13 min” Home practice: Attend to taste and satisfaction/enjoyment Home practice: Stop eating when moderately full; eat (buffet) Eat one snack or meal per day mindfully (repeated for all sessions, with increasing number of meals/snacks to be eaten mindfully per day)
6	Outer wisdom and integrated mindful eating meditation	NA	Guided meditation practice (10 min) Check-in on home meditation practice, food, and eating Nutrition Hourly energy balance Mindfully eating fruits and vegetables Integrated mindful eating meditation	General mindfulness meditation track (10 or 20 min) Eat fruits and vegetables mindfully Mindful eating meditation 10 min Mindful choice meditation 10 min
7	Savoring, pot luck meal, and quality over quantity challenge	Participants were invited to prepare any food and make it in a form of a buffet, different meal options: one healthy meal and another that may not be healthy but is preferred by the participant, which they would like to consume less frequently and in moderation	Guided meditation practice (10 min) Check-in on home meditation practice, food, and eating Mindful savoring meditation Pot lock meal Review of the “Quality over quantity” challenge	Choose among guided meditation (20 min, full instructions), guided mindfulness (minimal instructions), or guided mindful eating meditation tracks If general mindfulness (guided meditation or guided mindfulness) is chosen, choose to practice with or without the audio track
8	Mindful movement, chain analysis, and stress and eating	NA	Guided meditation practice (10 min) Check-in on home meditation practice, food, and eating Mindful movement/physical activity Walking meditation Chain analysis Stress and eating Eating trigger meditation Craving meditation Chair yoga	Choose among guided meditation (20 min, full instructions), guided mindfulness (minimal instructions), or guided mindful eating meditation tracks If general mindfulness (guided meditation or guided mindfulness) is chosen, choose to practice with or without the audio track
9	Chain analysis, personal values, and maintaining change	NA	Guided meditation practice (10 min) Check-in on home meditation practice, food, and eating Chain analysis Personal values exercise “My favorite food” meditation Maintaining change Brief closing meditation practice	Choose among guided meditation (20 min, full instructions), guided mindfulness (minimal instructions), or guided mindful eating meditation tracks If general mindfulness (guided meditation or guided mindfulness) is chosen, choose to practice with or without the audio track
10	Mindful snacking and KEEP in balance review	NA	Guided meditation practice (10 min) Check-in on home meditation practice, food, and eating Wisdom meditation Mindful snacking KEEP in balance review Self-acceptance meditation	Choose among guided meditation (20 min, full instructions), guided mindfulness (minimal instructions), or guided mindful eating meditation tracks If general mindfulness (guided meditation or guided mindfulness) is chosen, choose to practice with or without the audio track Home practice: Eat all meals and snacks mindfully

Participants received a series of audio tracks for the daily meditation practice, including general mindfulness meditation (with full instructions or minimal instructions), guided mindfulness meditation, and guided mindful eating tracks. The sessions were delivered through Zoom using “Focus mode,” in which participants were able to see the hosts’ videos and shared content but not the videos of other participants. Participants were assigned with participant number that they used to join the Zoom sessions. Therefore, no personal information was displayed in the Zoom meeting, and profile pictures were hidden. This strategy was adopted to create a safe, comfortable, and judgment-free environment for the participants and promote discussion during the sessions in which participants were invited to reflect on their eating habits, eating triggers, emotions, and situations that led them to overeat.

After each weekly session, participants answered a series of structured questions to reflect on their meditation practice and experience in the program. The questions asked during the program aimed to reflect on the content learnt, the mindfulness meditation practices, and the participants’ own experience in an attempt to improve their understanding of MB-EAT. Therefore, the reflections were not analyzed.

### Measures

Emotional eating was the primary outcome evaluated at pre-test, 5 weeks after the intervention started, and post-test using the Salzburg Emotional Eating Scale. Secondary outcomes, including response to emotions and emotion regulation strategies (Emotion Regulation Questionnaire), depression, anxiety, stress (Depression, Anxiety and Stress Scale), mindfulness (Mindful Eating Questionnaire and Mindful Attention Awareness Scale), mindful eating (Mindful Eating Questionnaire), weight, and body mass index (BMI), were evaluated at baseline and post-test. Daily meditation practice during the intervention period was self-reported using an online form. After each weekly session, participants answered structured questions online to reflect on their practice and experience in the program. Participants were contacted via email to complete questionnaires online.

## Emotional eating

*Salzburg Emotional Eating Scale* (SEES) measures changes in eating behavior (decreased or increased food intake, or no change in food intake when experiencing emotions) in response to specific emotions like happiness, sadness, anger, and anxiety (Meule et al., 2018a). This scale was used to measure emotional eating as the primary outcome. The SEES comprises four subscales with 5 items in each subscale (20-item scale; 0–100). The four subscales are positive (happy subscale), unpleasant but low-arousal emotions (sadness), unpleasant but high-arousal emotions (anger and anxiety), and response options range from 1 (I eat much less than usual) to 5 (I eat much more than usual). Higher scores suggest that an individual’s emotional states have a significant influence on their eating behavior. Specifically, higher scores indicate a greater tendency for emotions to lead to increased eating. Conversely, lower scores suggest that the individual eats less in response to emotions or relies less on food for emotional comfort. Good to excellent internal consistency has been reported for the scale (Cronbach’s *α* = 0.732–0.871) (Meule et al., 2018a).

*Dutch Eating Behavior Questionnaire* (DEBQ). DEBQ, specifically the emotional eating subscale (13 items), was administered during the recruitment phase to identify individuals with emotional eating (as indicated by a cutoff score ≥ 2.8) (Macht, 2022; van Strien et al., 1986). DEBQ assesses the eating in response to negative emotions using three subscales, including eating behaviors-dietary restraint, emotional eating, and external-based eating (van Strien et al., 1986). The Emotional Eating subscale measures overeating behaviors triggered by negative emotions, such as anger, boredom, anxiety, or fear. Each item had to be rated from 1 = never to 5 = very often. The average score of this subscale was calculated for comparison with the cutoff score. A higher score means a higher level of emotional eating. This questionnaire has demonstrated excellent internal consistency (Cronbach’s alpha (*α*) = 0.909) (Meule et al., 2018a).

## Eating behavior in response to stress and emotion regulation

*Salzburg Stress Eating Scale (SSES)* measures how people’s eating behavior changes in response to stress. It assesses whether they tend to eat less, eat the same amount, or eat more when they are stressed. The 10 items depict stressful situations, asking individuals how they react to such situations, with answers ranging from 1 (I eat much less than usual) to 5 (I eat much more than usual). Average scores are calculated, and higher values represent eating more when stressed and lower values represent eating less when stressed. Excellent internal consistency has been reported for the scale (Cronbach’s *α* = 0.899) (Meule et al., 2018b).

*Emotion Regulation Questionnaire* (ERQ) is a 10-item questionnaire based on Gross’s process model of emotion regulation (Gross & John, 2003). This model categorizes emotion regulation strategies based on how early they are activated in the emotion generation process and hypothesizes that different regulation strategies might have different consequences (Gross & John, 2003). The ERQ is designed to measure people’s usage of two regulation strategies, including an antecedent-focused strategy called Cognitive Reappraisal (6 items; e.g., “When I’m faced with a stressful situation, I make myself think about it in a way that helps me stay calm”) when a person attempts to change how he or she thinks about a situation to change its emotional impact, and a response-focused strategy called Expressive Suppression (4 items; e.g., “I keep my emotions to myself”) when a person attempts to inhibit the behavioral expression of his or her emotions. Separate scale scores are derived for these two regulation strategies. All items are answered on a 7-point Likert scale, ranging from 1 (strongly disagree) to 7 (strongly agree). The average of scores in each subscale represents how people use that emotion regulation strategy, with higher scores indicating greater usage of that strategy (Preece et al., 2020). The ERQ Cognitive Reappraisal and Expressive Suppression subscales have excellent (Cronbach’s *α* = 0.90) and good internal consistency, respectively (Cronbach’s *α* = 0.80) (Preece et al., 2020).

## Depression, anxiety, and stress

*Depression, Anxiety, and Stress Scale* (DASS-21) is a self-report scale that consists of 21 items aiming to evaluate negative emotional states of Depression (items 3, 5, 10, 13, 16, 17, and 21), Anxiety (items 2, 4, 7, 9, 15, 19, and 20), and Stress (items 1, 6, 8, 11, 12, 14, and 18). Each item is scored on a 4-point scale from 0 (did not apply to me at all) to 3 (applied to me most of the time). The total score for each subscale will be multiplied by two to obtain the final scores because the DASS-21 scale is a short-form version of the DASS-42 (42 items). Also, each subscale score will be calculated. A higher score on the subscales indicates greater severity or frequency of negative emotional symptoms (Henry & Crawford, 2005; Lovibond & Lovibond, 1995). In both longitudinal and cross-sectional studies of school teachers, all three subscales have demonstrated good to excellent internal consistency (Cronbach’s *α* = 0.86 to 0.92) (Cao et al., 2023).

## Mindfulness and mindful eating

*Mindful Attention Awareness Scale* (MAAS). MASS, a single-factor 15-item scale, that assesses the level of mindfulness of each participant. Participants who respond by indicating how frequently they have had the experience described in each statement using a 6-point Likert scale from 1 (almost always) to 6 (almost never). The minimum and maximum scores of participants on the scale are 15 to 90, respectively. The questionnaire has demonstrated good internal consistency (Cronbach’s *α* = 0.82) (Brown & Ryan, 2003).

*Mindful Eating Questionnaire* (MEQ) measures mindful eating using five subscales: Awareness (questions 10, 12, 16, 21, 20, 22, and 26), Distraction (questions 1, 6, and 28), Disinhibition (questions 2, 5, 7, 9, 11, 15, 18, and 25), Emotional Response (questions 13, 17, 19, and 27), and External Cues (questions 3, 4, 8, 14, 23, and 24). Each subscale score was calculated using the total points of that subscale divided by the number of items answered; the total score of MEQ is the mean of the total subscale scores. The eating behaviors are rated on a four-point Likert scale: 1—never/rarely, 2—sometimes, 3—often, and 4—usually/always. The Reverse scoring was applied to questions 1, 2, 6, 7, 9, 11, 17, 18, 19, 27, and 28 (Abdul Basir et al., 2021). This scale has acceptable internal consistency (Cronbach’s *α* = 0.64) (Framson et al., 2009). A higher total score indicates greater mindful eating. Higher scores in the Awareness and External Cues subscales indicate greater awareness (awareness of hunger and satiety signals as well as being present and engaged when eating) and greater awareness of external cues (awareness of responsiveness to environmental factors influencing eating behaviors). A higher score in the Distraction, Disinhibition, and Emotional Response subscales indicates lower distraction (the extent to which individuals eat while distracted), disinhibition (the individual’s ability to resist or inhibit the impulse to eat in response to environmental triggers), and emotional response (the extent to which individuals use food as a way to cope with emotional states).

*Record of daily mindfulness* practice during the intervention period was done using a daily online form to record the frequency, duration, and type of mindfulness practice (sitting meditation, guided or unguided meditation, and mini meditation) (Kristeller & Jordan, 2018). The durations of guided mindfulness practices using audio tracks were 6 min for the mini-meditation, and 10, 13, and 20 min for other mindfulness and mindful eating practices (Table 1). Participants could select multiple mindfulness practices if they used more than one guided audio track. If participants practiced mindfulness without an audio track, they specified the duration of their practice in minutes. The total meditation time in minutes per participant in the MB-EAT group was recorded for analysis.

## Anthropometric measures

*Weight and BMI.* Height and weight were measured in the laboratory using standardized procedures and used to calculate BMI = (weight (kg)/height (m)^2^).

### Statistical analysis

Descriptive analyses of baseline data were computed and reported as frequencies and percentages for categorical data, mean and standard deviation (SD) for continuous data. Normality for all outcome measures was determined using Shapiro-Wilks tests. Independent t or Mann–Whitney U tests were used to compare between-group differences at baseline according to continuous or ordinal levels of data, respectively. The assumption of sphericity for repeated measures ANOVA was checked using Mauchly’s test. Homogeneity of variance for between-group comparisons was assessed using Levene’s test. Outliers were identified through examination of boxplots and z-scores. Independent t-test or Mann–Whitney U tests were used to compare between-group differences at baseline, with homogeneity of variance checked using Levene’s test. When the normality assumption was not met, nonparametric tests were used. Standardized effect sizes for within- and between-group differences were reported as Cohen’s d with values from 0.00–0.20, 0.20–0.50, 0.50–0.80, and > 0.80 representing very small, small, medium, and large effect sizes, respectively (Portney & Watkins, 2017). A mixed analysis of variance (ANOVA) was performed to assess significant differences in emotional eating between the MB-EAT and the waitlist group at baseline, 5 weeks, and posttest. A mixed ANOVA was also used to analyze the differences in the other study variables between the MB-EAT and the waitlist group. Before the ANOVA test, the sphericity assumption was checked using Mauchly’s test; if violated, the Greenhouse–Geisser correction was applied. Effect sizes were reported as eta-squared (ηp2) values ranging from ≤ 0.01, 0.02–0.06, 0.07–0.14, and > 0.14, reflecting small, medium, large, and very large effect sizes, respectively (Richardson, 2011). Pearson’s correlation analysis or Spearman’s correlation with the Benjamini–Hochberg method was applied to control the False Discovery Rate (FDR) (Benjamini & Hochberg, 1995). The Benjamini–Hochberg method procedure is designed to reduce the likelihood of false positives when conducting multiple statistical tests, thereby increasing the reliability of the identified significant relationships (Benjamini & Hochberg, 1995). The analysis was conducted to identify relationships among variables at post-intervention that are relevant to emotional eating. The rationale was that certain psychosocial factors may influence eating behaviors. Variables were grouped into four categories: (1) eating behavior (emotional eating, stress eating), (2) mindfulness (mindfulness and mindful eating), (3) affective and stress factors (depression, anxiety, and stress), and (4) emotion regulation. The selected correlations are based on the hypothesis that emotional eating negatively correlates with mindfulness and mindful eating, suggesting that greater mindfulness may help reduce emotional eating. Specific facets of mindful eating are hypothesized to play a significant role in this relationship. Conversely, higher levels of depression, anxiety, and stress are hypothesized to positively correlate with emotional eating, indicating that increased negative affect and stress may contribute to stress-related eating behaviors. These analyses aim to identify potential psychological and behavioral factors that affect emotional eating post-intervention, providing insights into mechanisms underlying eating behaviors. Values of 0.0–0.2, 0.2–0.4, 0.4–0.6, 0.6–0.8, 0.8–1.0 represent very weak, weak, moderate, strong, or very strong correlations, respectively (Portney & Watkins, 2017). All data were analyzed using Datatab (DATATab team, 2025). Statistical significance was based on an alpha threshold of *p* < 0.05.

## Results

### Participants

**Fig. 1:**
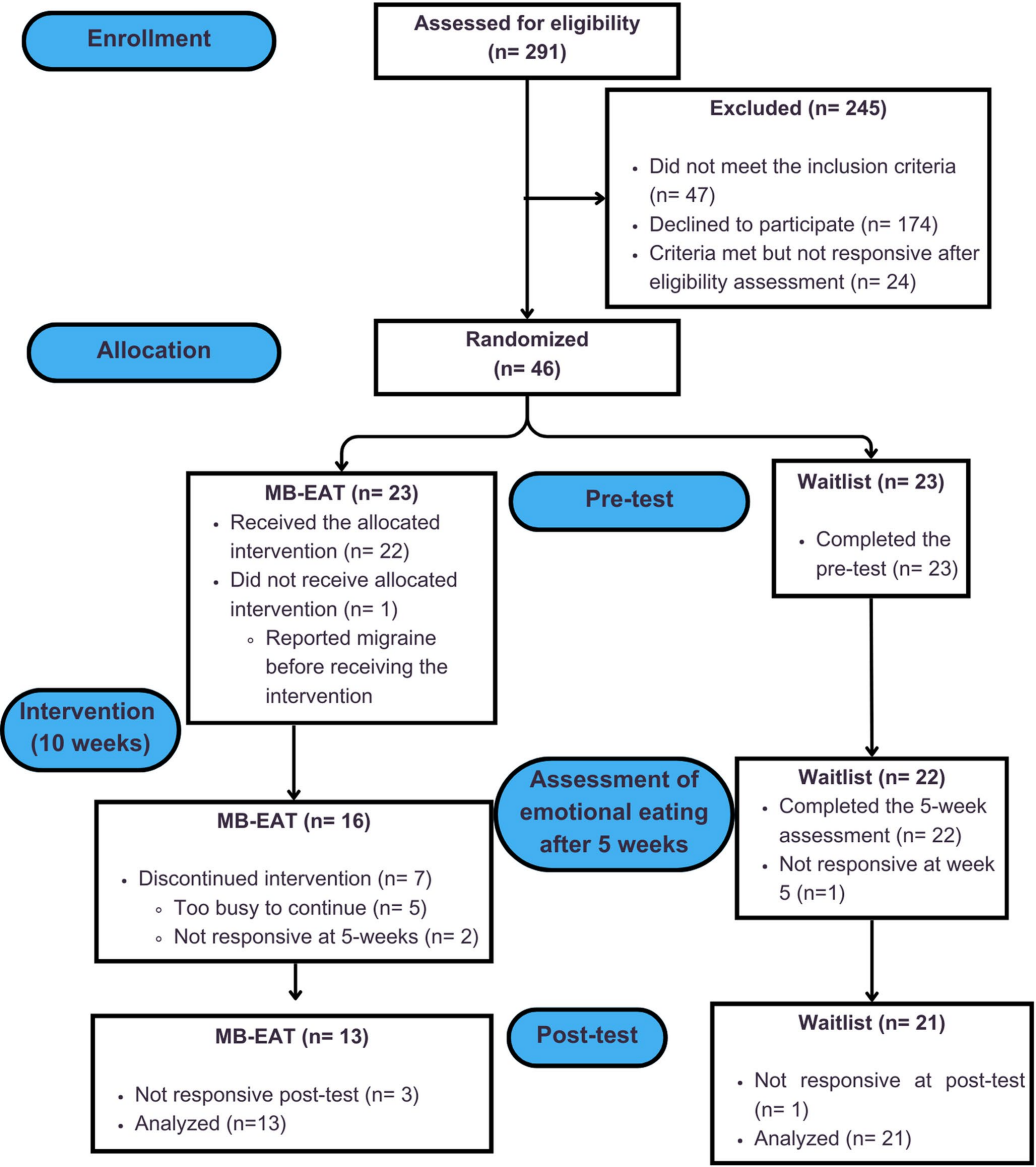
Consort chart

The workflow process, including recruitment, screening, allocation and pre-test, post-test analyses, is illustrated in Fig. 1. Briefly, a total of 291 participants were screened for eligibility, with 46 individuals meeting the criteria and subsequently randomized into either the MB-EAT group or the waitlist control group (Fig. 1). The mean age of participants in the MB-EAT group (*n* = 23) was 23.48 years, while it was 23.04 years in the waitlist group (*n* = 23) (Table 2). Both groups had the same sex distribution, with a ratio of 21 females to 2 males. In terms of BMI, 60% of the participants in the MB-EAT group (*n* = 14) were classified as having a normal BMI, while 8.7% (*n* = 2) were classified as overweight (BMI 23.0 to 24.9 kg/m^2^) and 30.4% (*n* = 7) as obese (BMI 25.0 and above). In the waitlist group, 69.6% (*n* = 16) were classified as having a normal BMI, while 8.7% (*n* = 2) were classified as overweight and 21.7% (*n* = 5) as obese. The emotional eating scores, measured with the emotional eating subscale of the DEBQ, were 3.70 in the MB-EAT group and 3.67 in the waitlist group, both meeting the eligibility criterion of a score ≥ 2.8. At baseline, there were no statistically significant differences between the groups in terms of weight, BMI, eating behaviors (emotional and stress eating), emotion regulation, depression, anxiety, stress, mindfulness, or mindful eating scores (Table 2).

**Table 2:** Study variables at baseline

Variable	MB-EAT (*n* = 23)		Waitlist (*n* = 23)		*p*
	Mean	SD	Mean	SD	
Age^a^	23.48	4.02	23.04	3.78	0.768
Sex (F:M)	21:2		21:2		
Weight (kg) ^a^	62.97	10.21	58.44	11.54	0.073
Height (cm)^a^	163.30	4.39	160.78	7.15	0.189
BMI (kg/m^2^)^a^	23.60	3.61	22.69	3.82	0.260
DEBQ: Emotional eating^b^	3.70 (range: 3.00–4.80)	0.49	3.67 (range: 2.80–4.92)	0.57	0.813
Emotional eating (SEES)^b^	3.44	0.52	3.24	0.57	0.220
Stress eating (SSES)^b^	3.62	0.85	3.29	0.90	0.208
Emotion regulation. ERQ: Cognitive appraisal^a^	4.63	1.01	4.91	0.81	0.347
Emotion regulation. ERQ: Expressive suppression^a^	4.03	1.30	4.18	1.32	0.662
DASS-21: Depression^a^	10.09	8.85	11.48	10.02	0.726
DASS-21: Anxiety^a^	10.6	6.33	12.52	6.61	0.336
DASS-21: Stress^b^	16.70	8.75	18.78	8.69	0.421
Mindful attention awareness (MAAS)^b^	3.39	0.91	3.17	0.78	0.382
Mindful eating. MEQ, total score^b^	2.30	0.27	2.36	0.29	0.438
Mindful eating. MEQ: Disinhibition^a^	2.00	0.46	2.17	0.54	0.233
Mindful eating. MEQ: Awareness^b^	2.45	0.50	2.66	0.65	0.227
Mindful eating. MEQ: External Cues^a^	2.79	0.46	2.53	0.38	0.086
Mindful eating. MEQ: Distraction^b^	2.45	0.55	2.48	0.65	0.870
Mindful eating. MEQ: Emotional response^b^	1.82	0.52	1.96	0.46	0.334

Values are reported as mean ± SD. ^a^Mann-Whitney U test; ^b^Independent T-test. *p* ≤ 0.05 statistically significant between group difference. Abbreviations: DASS-21, Depression, anxiety, and stress scale 21-item scale; ERQ, Emotion regulation questionnaire; MAAS, Mindful attention awareness scale; MEQ, Mindful eating questionnaire; SEES, Salzburg emotional eating scale; SSES, Salzburg stress eating scale

The baseline measures, shown in Table 2, compare various demographic and psychological variables between the MB-EAT group and the waitlist control group. Overall, there are no significant differences between the groups across these measures, as indicated by the p-values all being above 0.05. This suggests that the groups are well-matched at baseline. Key points include similar ages, sex distribution, weight, height, BMI, and measures of emotional eating, stress, emotion regulation, depression, anxiety, stress, mindfulness, and mindful eating. Also, no significant correlations among the demographic variables at baseline were found.

Five weeks after the commencement of the intervention, emotional eating was reassessed in both groups. Sixteen participants in the MB-EAT group (69.6%) and 22 participants in the waitlist group (95.7%) completed the assessment. At the post-test assessment, 13 participants in the MB-EAT group (56.5%) and 21 participants in the waitlist group (91.3%) completed the post-test assessment. A summary of baseline outcomes and participant characteristics is provided in Table 2. Attendance throughout the 10-week program showed a declining trend. The highest attendance was observed during sessions 1 to 4, with rates of 96%, 87%, 83%, and 83%, respectively. Attendance decreased to 61% in session 6, and then dropped further to 35% in session 7, remaining between 26 and 30% in the remaining sessions. The periods of low attendance coincided with examination periods at the university, suggesting that academic commitments may have impacted participants' ability to attend. By week 5 and end of the study, 13 participants remained, indicating a retention rate of approximately 56.5%, which reflects the overall decline in attendance but also highlights participant retention despite decreasing engagement over time.

The total meditation time reported by participants varied considerably, with a mean of 307 min (SD = 163.81), ranging from 39 to 632 min across 15 participants. Approximately 65.2% of participants (15 out of 23) reported their mindfulness practice. During each MB-EAT session, the mindfulness teacher inquired about participants' practice, and some reported forgetting to record their practice despite practicing regularly. Others indicated they practiced without using the audio track and lost track of time, resulting in incomplete recording of total practice time. Some used the audio tracks but failed to report their daily practice, either forgetting or intentionally omitting the data. Despite receiving email reminders, the practice reporting link, and ongoing reminders from the mindfulness teacher, feedback during sessions suggested that reporting was often inaccurate. This limitation in self-reported data affects the reliability of the total meditation time, leading us to exclude this outcome from the analysis.

### Mixed ANVOVA

As shown in Table 3, there was no significant group difference in emotional eating measured using the SEES. However, a significant change over time was observed, as well as a notable interaction between time and group. Post-hoc analysis showed that within the MB-EAT group, emotional eating scores decreased from pre-test to post-test, indicating an improvement over the course of the intervention.

**Table 3:** Descriptive statistics and the mixed ANOVA results on emotional eating score measured with the SEES at baseline, 5 weeks, and post-test

Group time	Groups			Mixed ANOVA			
	MB-EAT (*n* = 13)	Waitlist (*n* = 21)		Group	Time	Interaction	Post-hoc for interaction (Bonferroni)
	M ± SD	M ± SD					
Emotional eating (SEES) at pre-test	3.50 ± 0.56	3.18 ± 0.57		F(1,32) = 0.06 *p* = 0.806 ω^2^_p_ = 0.000	F(2,64) = 8.94*** ***p*** **<** **0.001** **ω**^**2**^_**p**_** = 0.190**	F(2,64) = 6.96** ***p*** **=** **0.002** **ω**^**2**^_**p**_** = 0.150**	**MB-EAT** Pre-test vs week 5 *p* = 0.001 Pre-test vs post-test *p* = 0.001 Week 5 vs post-test *p* = 0.874
Emotional eating (SEES) at week 5	3.07 ± 0.50	3.08 ± 0.62					
Emotional eating (SEES) at post-test	2.95 ± 0.43	3.12 ± 0.63					

*p < 0.05; **p < 0.01; ***p < 0.001. Statistically significant difference shown in bold

Table 4 shows the mixed ANOVA results for the other study variables. No significant differences between groups were found. Significantly large time effects were observed for stress eating, mindful attention awareness, and various aspects of mindful eating, including total mindful eating, Disinhibition, External Cues, and Emotional Response. These results suggest that there were no baseline differences between groups, and the significant time effects observed reflect changes over time rather than group differences at baseline. Most importantly, there were large and significant interaction effects for stress eating, mindful eating (total, Disinhibition, Awareness, and Distraction). Post-hoc analysis revealed that, within the MB-EAT group, stress eating scores decreased from pre-test to post-test. For mindful eating, the MB-EAT group showed an increase from pre-test to post-test, with post-test scores higher than those of the waitlist group. Similar patterns were observed in Disinhibition, Awareness, and Distraction subscales of mindful eating, with the MB-EAT group showing improvements over time compared to the waitlist group. These findings suggest that the MB-EAT intervention had a positive impact on reducing certain eating behaviors and enhancing mindful eating practices, as detailed in Table 4.

**Table 4:** Descriptive statistics and the mixed ANOVA results for the other study variables

Study variables	Group time	Groups		Mixed ANOVA			
		MB-EAT (*n* = 13)	Waitlist (*n* = 21)	Group	Time	Interaction	Post-hoc for interaction (Bonferroni)
		M ± SD	M ± SD				
Stress eating (SSES)	Pre-test	3.54 ± 0.87	3.22 ± 0.84	F(1,30) = 0.003 ω^2^_p_ = 0.000	F(1,30) = 5.662* ***p* = 0.024** **ω**^**2**^_**p**_** = 0.159**	F(1,30) = 7.338* ***p*** **=** **0.011** **ω**^**2**^_**p**_** = 0.196**	**MB-EAT:** Pre-test > Post-test
	Post-test	2.97 ± 0.52	3.26 ± 0.77				
Emotion regulation. ERQ: Cognitive Appraisal	Pre-test	4.50 ± 1.05	5.01 ± 0.59	F(1,30) = 1.004 ω^2^_p_ = 0.033	F(1,30) = 0.076 ω^2^_p_ = 0.002	F(1,30) = 2.623 ω^2^_p_ = 0.080	–
	Post-test	4.81 ± 0.78	4.79 ± 0.85				
Emotion regulation. ERQ: Expressive Suppression	Pre-test	3.85 ± 1.31	4.05 ± 1.35	F(1,30) = 0.381 ω^2^_p_ = 0.012	F(1,30) = 0.019 ω^2^_p_ = 0.000	F(1,30) = 0.185 ω^2^_p_ = 0.006	–
	Post-test	3.77 ± 1.00	4.09 ± 1.25				
DASS-21: Depression	Pre-test	10.09 ± 8.85	11.48 ± 10.02	F(1,30) = 1.906 ω^2^_p_ = 0.060	F(1,30) = 3.391 ω^2^_p_ = 0.101	F(1,30) = 1.520 ω^2^_p_ = 0.048	–
	Post-test	5.08 ± 7.86	10.67 ± 10.34				
DASS-21: Anxiety	Pre-test	10.26 ± 6.33	12.52 ± 6.61	F(1,30) = 0.160 ω^2^_p_ = 0.005	F(1,30) = 0.227 ω^2^_p_ = 0.007	F(1,30) = 0.056 ω^2^_p_ = 0.001	–
	Post-test	11.69 ± 4.75	11.71 ± 6.76				
DASS-21: Stress	Pre-test	16.70 ± 8.75	18.78 ± 8.69	F(1,30) = 1.651 ω^2^_p_ = 0.052	F(1,30) = 1.318 ω^2^_p_ = 0.043	F(1,30) = 1.461 ω^2^_p_ = 0.046	–
	Post-test	13.85 ± 5.51	18.1 ± 8.28				
Mindful attention awareness (MAAS)	Pre-test	3.35 ± 0.88	3.14 ± 0.75	F(1,30) = 3.619 ω^2^_p_ = 0.108	F(1,30) = 4.846* ***p*** **= 0.036** **ω**^**2**^_**p**_** = 0.139**	F(1,30) = 3.373 ω^2^_p_ = 0.101	–
	Post-test	3.90 ± 0.64	3.19 ± 0.80				
Mindful eating. MEQ, total score	Pre-test	2.31 ± 0.24	2.38 ± 0.28	F(1,30) = 1.951 ω^2^_p_ = 0.061	F(1,30) = 16.380*** ***p*** **<** **0.001** **ω**^**2**^_**p**_** = 0.353**	F(1,30) = 20.8*** ***p* < 0.001** **ω**^**2**^_**p**_** = 0.410**	**MB-EAT:** Pre-test < Post-test **Post-test:** MB-EAT > Waitlist
	Post-test	2.67 ± 0.23	2.36 ± 0.27				
Mindful eating. MEQ: Disinhibition	Pre-test	2.01 ± 0.41	2.23 ± 0.53	F(1,30) = 0.595 ω^2^_p_ = 0.019	F(1,30) = 30.519*** ***p*** **<** **0.001** **ω**^**2**^_**p**_** = 0.504**	F(1,30) = 17.57*** ***p* < 0.001** **ω**^**2**^_**p**_** = 0.369**	**MB-EAT:** Pre-test < Post-test **Post-test:** MB-EAT > Waitlist
	Post-test	2.78 ± 0.42	2.33 ± 0.50				
Mindful eating. MEQ: Awareness	Pre-test	2.43 ± 0.47	2.66 ± 0.71	F(1,30) = 0.018 ω^2^_p_ = 0.000	F(1,30) = 0.300 ω^2^_p_ = 0.009	F(1,30) = 5.672* ***p* = 0.024** **ω**^**2**^_**p**_** = 0.159**	**MB-EAT:** Pre-test < Post-test
	Post-test	2.68 ± 0.49	2.50 ± 0.64				
Mindful eating. MEQ: External Cues	Pre-test	2.73 ± 0.29	2.53 ± 0.39	F(1,30) = 1.911 ω^2^_p_ = 0.060	F(1,30) = 7.579** ***p*** **=** **0.010** **ω**^**2**^_**p**_** = 0.202**	F(1,30) = 0.380 ω^2^_p_ = 0.012	─
	Post-test	2.44 ± 0.41	2.34 ± 0.42				
Mindful eating. MEQ: Distraction	Pre-test	2.44 ± 0.52	2.54 ± 0.62	F(1,30) = 0.880 ω^2^_p_ = 0.028	F(1,30) = 2.303 ω^2^_p_ = 0.071	F(1,30) = 5.138* ***p* = 0.031** **ω**^**2**^_**p**_** = 0.146**	**MB-EAT:** Pre-test < Post-test **Post-test:** MB-EAT > Waitlist
	Post-test	2.87 ± 0.32	2.46 ± 0.63				
Mindful eating. MEQ: Emotional response	Pre-test	1.94 ± 0.53	1.95 ± 0.48	F(1,30) = 1.770 ω^2^_p_ = 0.056	F(1,30) = 14.904*** ***p*** **<** **0.001** **ω**^**2**^_**p**_** = 0.332**	F(1,30) = 3.248 ω^2^_p_ = 0.098	–
	Post-test	2.56 ± 0.45	2.17 ± 0.53				

*p < 0.05; **p < 0.01; ***p < 0.001

DASS-21, Depression, anxiety, and stress scale 21-item scale; ERQ, Emotion regulation questionnaire; MAAS, Mindful attention awareness scale; MEQ, Mindful eating questionnaire; SEES, Salzburg Emotional Eating Scale; SSES, Salzburg stress eating scale. Statistically significant difference shown in bold

### Between-group comparisons

Participants in the MB-EAT group showed significantly lower levels of depression compared to those in the waitlist group (Table 5), indicating that the intervention was associated with a reduction in depressive symptoms. In terms of mindful eating, participants in the MB-EAT group showed greater overall mindful eating, reflecting increased present-moment awareness during eating. Specifically, the MB-EAT group exhibited improved regulation over disinhibited eating behaviors, indicating a greater ability to resist environmental triggers that might lead to overeating. Additionally, participants in the MB-EAT group were less prone to distracted eating, suggesting improved focus and attention during meals. Finally, after the MB-EAT intervention, participants reported a lower tendency to use food as a coping mechanism for emotional distress, as observed in a higher Emotional Response score in the MEQ, highlighting improved emotional regulation related to eating behaviors. These findings underscore the positive impact of the MB-EAT program on reducing depressive symptoms and enhancing several aspects of mindful eating.

**Table 5:** Study variables at post-test

Variable	MB-EAT (*n* = 13)		Waitlist (*n* = 21)		*p*	Effect size (Cohen's d)
	Mean	SD	Mean	SD		
Weight (kg)^a^	63.76	10.81	58.09 (*n* = 20)	10.84	0.087	0.63
BMI (kg/m^2^)^a^	23.74	3.69	22.45 (*n* = 20)	3.73	0.298	0.39
Emotional eating (SEES)^b^	2.95	0.43	3.12	0.63	0.384	0.31
Stress eating (SSES)^b^	3.01	0.52	3.26 (*n* = 20)	0.77	0.256	0.41
Emotion regulation. ERQ: Cognitive Appraisal^a^	4.86	0.78	4.75 (*n* = 20)	0.85	1.000	0.00
Emotion regulation. ERQ: Expressive Suppression^b^	3.73	1.03	4.09 (*n* = 20)	1.22	0.438	0.28
DASS-21: Depression^a^	5.08	7.86	10.67	10.34	**0.036**	**0.80**
DASS-21: Anxiety^a^	11.69	4.75	11.71	6.76	0.738	0.12
DASS-21: Stress^b^	13.85	5.51	18.10	8.28	0.112	0.58
Mindful attention awareness (MAAS)^b^	3.91	0.66	3.19 (n = 20)	0.80	0.054	0.71
Mindful eating. MEQ, total score^a^	2.70	0.21	2.36 (*n* = 20)	0.27	**0.004**	**1.09**
Mindful eating. MEQ: Disinhibition^a^	2.85	0.36	2.33 (*n* = 20)	0.50	**0.008**	**1.04**
Mindful eating. MEQ: Awareness^b^	2.73	0.49	2.50 (*n* = 20)	0.64	0.463	0.26
Mindful eating. MEQ: External Cues^b^	2.46	0.42	2.34 (*n* = 20)	0.42	0.484	0.25
Mindful eating. MEQ: Distraction^a^	2.89	0.33	2.50 (*n* = 20)	0.64	0.102	0.61
Mindful eating. MEQ: Emotional response^b^	2.58	0.46	2.17 (*n* = 20)	0.53	**0.043**	**0.75**

Values are reported as mean ± SD. ^a^Mann-Whitney U test; ^b^Independent T-test. *p* ≤ 0.05 statistically significant between group difference.  ADASS-21, Depression, anxiety, and stress scale 21-item scale; ERQ, Emotion regulation questionnaire; MAAS, Mindful attention awareness scale; MEQ, Mindful eating questionnaire; SEES, Salzburg emotional eating scale; SSES, Salzburg stress eating scale. Statistically significant difference shown in bold

### Correlation analysis

The correlation analysis conducted at post-test aimed to explore the relationships among various psychosocial factors and eating behaviors within the MB-EAT group and waitlist control, focusing on how these variables interact to influence emotional and stress-related eating. Several significant correlations were observed at post-test in the MB-EAT group (*n* = 13, Table 6). Emotional eating was strongly positively correlated with Cognitive Appraisal as measured by the ERQ (*r* = 0.840, *p* < 0.001). This indicates that individuals with higher emotional eating tendencies tend to have greater cognitive appraisal, suggesting they may engage more in evaluative thinking related to their emotions. A robust positive correlation was found between Cognitive Appraisal and stress eating (*r* = 0.790, *p* < 0.001), implying that higher levels of cognitive appraisal are associated with increased stress-related eating behaviors, suggesting that individuals who are more engaged in cognitive evaluation may be more prone to eating in response to stress. Furthermore, Depression was strongly negatively correlated with the Awareness subscale of the MEQ (*ρ* = −0.80, *p* < 0.001). This indicates that higher depressive symptoms are associated with lower awareness during eating, suggesting that individuals experiencing greater depression may be less attentive or conscious of their eating processes, potentially leading to overeating.

**Table 6:** The combined Pearson’s and Spearman’s correlation matrix among eating behavior, mindfulness, affective factors, stress, and emotion regulation in the MB-EAT group (*n* = 13) at post-test

Variables	Salzburg emotional eating scale	Salzburg stress eating scale	Emotion regulation questionnaire: cognitive appraisal	Emotion regulation questionnaire: cognitive appraisal	Depression, anxiety, and stress scale: depression	Depression, anxiety, and stress scale: anxiety	Depression, anxiety, and stress scale: stress	Mindful attention awareness scale	Mindful eating questionnaire: total	Mindful eating questionnaire: disinhibition	Mindful eating questionnaire: awareness	Mindful eating questionnaire: external cues	Mindful eating questionnaire: distraction	Mindful eating questionnaire: emotional response
Salzburg emotional eating scale	–													
Salzburg stress eating scale	0.910^a#^ *p* < 0.001	–												
Emotion regulation questionnaire: cognitive appraisal	**0.840**^**a**^ ***p* < 0.001**	**0.790**^**a**^ ***p* < 0.001**	–											
Emotion regulation questionnaire: cognitive appraisal	−0.180^a^ *p* = 0.559	−0.350^a^ *p* = 0.248	−0.250^a^ *p* = 0.408	–										
Depression, anxiety, and stress scale: depression	−0.390^b^ *p* = 0.192	−0.420^b^ *p* = 0.157	−0.380^b^ *p* = 0.197	0.440^b^ *p* = 0.131	–									
Depression, anxiety, and stress scale: anxiety	−0.540^b^ *p* = 0.058	−0.550^b^ *p* = 0.05 0	−0.460^b^ *p* = 0.117	0.320^b^ *p* = 0.288	0.600^b #^ *p* = 0.030	–								
Depression, anxiety, and stress scale: stress	−0.090^a^ *p* = 0.760	−0.190^a^ *p* = 0.530	−0.230^a^ *p* = 0.453	0.650^a #^ *p* = 0.016	0.060^b^ *p* = 0.842	0.380^b^ *p* = 0.195	–							
Mindful attention awareness scale	−0.040^a^ *p* = 0.895	−0.090^a^ *p* = 0.779	−0.140^a^ *p* = 0.651	−0.300^a^ *p* = 0.323	−0.350^b^ *p* = 0.239	−0.350^b^ *p* = 0.243	−0.470^a^ *p* = 0.104	–						
Mindful eating questionnaire: total	−0.120^a^ *p* = 0.702	−0.120^a^ *p* = 0.695	0.070^a^ *p* = 0.809	−0.390^a^ *p* = 0.192	−0.640^b #^ *p* = 0.018	−0.360^b^ *p* = 0.233	−0.160^a^ *p* = 0.601	0.400^a^ p = 0.180	–					
Mindful eating questionnaire: disinhibition	0.150^b^ *p* = 0.627	0.150^b^ *p* = 0.622	0.210^b^ *p* = 0.494	0.020^b^ *p* = 0.948	−0.580^b #^ *p* = 0.036	−0.290^b^ *p* = 0.345	0.380^b^ *p* = 0.202	−0.200^b^ *p* = 0.502	0.610^b #^ *p* = 0.026	–				
Mindful eating questionnaire: awareness	0.510^a^ *p* = 0.072	0.490^a^ *p* = 0.086	0.50^a^ *p* = 0.083	−0.360^a^ *p* = 0.23	−**0.80**^**b**^ ***p* < 0.001**	−0.350^b^ *p* = 0.244	−0.150^a^ *p* = 0.623	0.520^a^ *p* = 0.067	0.400^a^ *p* = 0.181	0.530^b^ *p* = 0.06	–			
Mindful eating questionnaire: external cues	−0.400^a^ *p* = 0.176	−0.180^a^ *p* = 0.553	−0.30^a^ *p* = 0.321	−0.160^a^ *p* = 0.611	0.190^b^ *p* = 0.524	0.310^b^ *p* = 0.301	−0.050^a^ *p* = 0.864	0.030^a^ *p* = 0.929	0.310^a^ *p* = 0.308	−0.190^b^ *p* = 0.533	−0.350^a^ *p* = 0.244	–		
Mindful eating questionnaire: distraction	−0.190^a^ *p* = 0.538	−0.330^a^ *p* = 0.274	−0.240^a^ *p* = 0.427	−0.160^a^ *p* = 0.592	−0.410^b^ *p* = 0.161	−0.460^b^ *p* = 0.116	0.050^a^ *p* = 0.881	0.303^a^ *p* = 0.269	0.680^a #^ *p* = 0.010	0.420^b^ *p* = 0.150	0^a^ *p* = 0.990	0.180^a^ *p* = 0.562	–	
Mindful eating questionnaire: emotional response	−0.410^a^ *p* = 0.165	−0.500^a^ *p* = 0.085	−0.120^a^ *p* = 0.708	−0.240^a^ *p* = 0.437	−0.150^b^ *p* = 0.625^b^	−0.080^b^ *p* = 0.796	−0.350^a^ *p* = 0.235	0.310^a^ *p* = 0.302	0.750^a #^ *p* = 0.003	0.170^b^ *p* = 0.572	−0.070^a^ *p* = 0.816	0.250^a^ *p* = 0.417	0.590^a #^ *p* = 0.034	-

^a^*p* ≤ 0.05 statistically significant correlation (Pearson’s r)

^b^*p* ≤ 0.05 statistically significant correlation (Spearman’s ρ)

Statistically significant correlations are shown in bold

^#^Not significant after applying the Benjamini–Hochberg method

The Benjamini–Hochberg method was used for controlling the False Discovery Rate (Q = 0.05, global correction)

In the waitlist group at post-test (*n* = 21, Table 7), there was a strong negative correlation between stress eating and Emotional Response measured by the MEQ (*r* = −0.770, *p* < 0.001). This association suggests that higher stress eating is linked to higher emotional response. Also,

**Table 7:** The combined Pearson’s and Spearman’s correlation matrix among eating behavior, mindfulness, affective factors, stress, and emotion regulation in the waitlist group (*n* = 21) at post-test

Variables	Salzburg emotional eating scale	Salzburg stress eating scale	Emotion regulation questionnaire: cognitive appraisal	Emotion regulation questionnaire: cognitive appraisal	Depression, anxiety, and stress scale: depression	Depression, anxiety, and stress scale: anxiety	Depression, anxiety, and stress scale: stress	Mindful attention awareness scale	Mindful eating questionnaire: total	Mindful eating questionnaire: disinhibition	Mindful eating questionnaire: awareness	Mindful eating questionnaire: external cues	Mindful eating questionnaire: distraction	Mindful eating questionnaire: emotional response
Salzburg emotional eating scale	–													
Salzburg stress eating scale	0.860^a#^ *p* < 0.001	–												
Emotion regulation questionnaire: cognitive appraisal	−0.110^b^ *p* = 0.668	−0.030^b^ *p* = 0.906	–											
Emotion regulation questionnaire: cognitive appraisal	0.190^a^ *p* = 0.437	0.490^a #^ *p* = 0.031	−0.300^a^ *p* = 0.216	–										
Depression, anxiety, and stress scale: depression	0.110^b^ *p* = 0.640	0.060^b^ *p* = 0.808	−0.320^b^ *p* = 0.184	0.160^b^ *p* = 0.500	–									
Depression, anxiety, and stress scale: anxiety	0.130^a^ *p* = 0.606	0.350^a^ *p* = 0.136	−0.330^a^ *p* = 0.166	0.350^a^ *p* = 0.140	0.480^b #^ *p* = 0.040	–								
Depression, anxiety, and stress scale: stress	0.270^a^ *p* = 0.264	0.260^a^ *p* = 0.290	−0.200^a^ *p* = 0.404	0.160^a^ *p* = 0.520	0.430^b^ *p* = 0.063	0.540^a #^ *p* = 0.016	–							
Mindful attention awareness scale	−0.150^a^ *p* = 0.542	−0.220^a^ *p* = 0.375	−0.170^a^ *p* = 0.486	−0.050^a^ *p* = 0.848	−0.350^b^ *p* = 0.146	−0.50^a #^ *p* = 0.029	**−0.730**^**a**^ ***p* < 0.001**	–						
Mindful eating questionnaire: total	−0.390^b^ *p* = 0.101	−0.590^b #^ *p* = 0.008	−0.190^b^ *p* = 0.428	−0.050^b^ *p* = 0.825	0.230^b^ *p* = 0.353	−0.260^b^ *p* = 0.284	−0.010^b^ *p* = 0.963	0.150^b^ *p* = 0.527	–					
Mindful eating questionnaire: disinhibition	−0.330^a^ *p* = 0.170	−0.360^a^ *p* = 0.136	−0.540^a #^ *p* = 0.016	0.280^a^ *p* = 0.249	0.570^b #^ *p* = 0.012	0.210^a^ *p* = 0.378	0.030^a^ *p* = 0.903	0.310^a^ *p* = 0.192	0.490^b #^ *p* = 0.032	–				
Mindful eating questionnaire: awareness	−0.200 ^a^ *p* = 0.409	−0.170^a^ *p* = 0.474	0^a^ *p* = 0.995	0.020^a^ *p* = 0.921	−0.140^b^ *p* = 0.564	0.030^a^ *p* = 0.911	0.030^a^ *p* = 0.894	0^a^ *p* = 1	0.570^b #^ *p* = 0.011	0.100^a^ *p* = 0.694	–			
Mindful eating questionnaire: external cues	0.290^a^ *p* = 0.228	0.140^a^ *p* = 0.558	0.120^a^ *p* = 0.626	−0.050^a^ *p* = 0.842	0.120^b^ *p* = 0.625	−0.190^a^ *p* = 0.433	0.310^a^ *p* = 0.191	−0.250^a^ *p* = 0.308	0.330^b^ *p* = 0.170	0.040^a^ *p* = 0.857	0.100^a^ *p* = 0.672	–		
Mindful eating questionnaire: distraction	−0.220^a^ *p* = 0.357	−0.310^a^ *p* = 0.193	−0.100^a^ *p* = 0.671	−0.130^a^ *p* = 0.592	−0.210^b^ *p* = 0.392	−0.500^a #^ *p* = 0.029	−0.550^a #^ *p* = 0.015	0.490^a #^ *p* = 0.032	0.380^b^ *p* = 0.106	−0.020^a^ *p* = 0.937	0.150^a^ *p* = 0.536	−0.180^a^ *p* = 0.465	–	
Mindful eating questionnaire: emotional response	−0.590 ^a #^ *p* = 0.007	**−0.770**^**a**^ ***p* < 0.001**	−0.050^a^ *p* = 0.839	−0.350^a^ *p* = 0.140	−0.130^b^ *p* = 0.599	−0.390^a^ *p* = 0.100	−0.400^a^ *p* = 0.094	0.400^a^ *p* = 0.089	0.490^b #^ *p* = 0.034	0.350^a^ *p* = 0.140	−0.110^a^ *p* = 0.655	−0.180^a^ *p* = 0.451	0.270^a^ *p* = 0.270	–

^a^*p* ≤ 0.05 statistically significant correlation (Pearson’s r)

^b^*p* ≤ 0.05 statistically significant correlation (Spearman’s ρ)

Statistically significant correlations are shown in bold

^#^Not significant after applying the Benjamini–Hochberg method

The Benjamini–Hochberg method was used for controlling the False Discovery Rate (Q = 0.05, global correction)

 a strong negative correlation was found between stress and mindfulness (*r* = −0.730, *p* < 0.001), indicating that higher levels of stress are associated with lower levels of mindfulness.

These findings reveal that, in the MB-EAT group, higher emotional eating is strongly associated with increased cognitive appraisal, and lower awareness is linked to greater depression, supporting the hypothesis that emotional regulation and mindfulness are key factors influencing eating behaviors. Conversely, in the waitlist group, stress and emotional responses are inversely related to mindfulness, highlighting the potential role of mindfulness in mitigating stress-related eating. Overall, these differences suggest that the MB-EAT intervention may enhance emotional regulation and mindfulness, contributing to reductions in emotional and stress eating behaviors.

## Discussion

This randomized controlled trial aimed to evaluate the effectiveness of MB-EAT on emotional eating in university students classified with emotional eating behaviour. Our findings show a trend towards a reduction in emotional eating scores between those in the MB-EAT intervention when compared to the waitlist control group at the post-test period. Significant time and time-by-group interaction effect were identified. Individuals classified with emotional eating who received MB-EAT showed a reduction in depression scores and increased mindful eating skills. The findings of the correlation analysis indicate a strong association between mindful eating ability, emotional regulation and emotional eating behavior in the MB-EAT group.

Emotional eating is a multifaceted issue that is highly prevalent across various populations and leads to poor psychological and physical health. Usually, emotion-induced overeating elicits negative emotions, and vice versa, which worsens over time. Emotional eating leads to a vicious, reiterative cycle of negative affect and overeating that worsens over time. This cycle increases the risk of developing binge eating and cardiometabolic disease. In addition, the interactions between eating behavior and emotion hinder the development of effective treatment and prevention strategies.

The significant effect of time on emotional eating implies that the duration of the intervention plays a crucial role in the improvement of emotion regulation among individuals with emotional eating. However, there are negative effects of time, which makes people shift between different subtypes of emotional eating and results in aggravation of emotional eating (Xu et al., 2024). Of note, the mean emotional eating scores of the MB-EAT group measured at three-time points are indicative of a decreasing trend that exhibited larger and more sustained reductions in the emotional eating score when compared to the waitlist group. However, they did not result in a significant group difference. In addition, MB-EAT strongly interacts with time underlying an accumulative effect that can reinforce the effectiveness of MB-EAT.

Our findings align with previous evidence that suggests MB-EAT holds promise in reducing emotional eating. A previous study that also used a 10-week mindful eating intervention demonstrated an interaction between mindfulness and time in emotional eating in women with overweight classified with emotional eating behavior (Salvo et al., 2022). However, it is possible that an intervention of longer duration would lead to significantly reduced levels of emotional eating over time (Daniela Mercado et al., 2021; Warren et al., 2017). Given functional magnetic resonance imaging (fMRI) has revealed that naïve and adept mindfulness practitioners can regulate emotion through distinct neural mechanisms (Zeidan et al., 2019), we speculate MB-EAT can equip individuals with emotional eating with different strategies to regulate emotion boosting the stability of eating behavior over time.

The significant interaction effect between mindful eating skills and time and increased mindful eating ability at post-test underpins the effect of MB-EAT; students did progressively get alleviation of their emotional eating over time, suggesting that consistent engagement with mindfulness techniques can lead to lasting changes. Besides mindful eating ability, the improvement of psychological state may contribute to the sustained effect of this study.

The correlation analyses provided valuable insights into the relationships between emotional eating and other psychological factors. In the MB-EAT group, strong positive correlations among emotional eating, stress eating, and emotional regulation were found as well as a strong negative correlation between depression and the awareness subscale in mindful eating. The relationship between eating behaviors and disorders with cognitive reappraisal, an adaptative strategy that involves changing the way one is thinking about a situation to modify its emotional impact, has not been consistent (Larionow et al., 2025; Salias et al., 2025). For instance, cognitive reappraisal has been considered an adaptative strategy (Salias et al., 2025). However, a weak correlation between the use of adaptative strategies such as cognitive reappraisal and disordered eating symptoms has been reported as weak (Salias et al., 2025) suggesting that more research is needed to understand the role of emotion regulation in disordered eating. A strong positive correlation between cognitive reappraisal and emotional and stress eating was found which indicates that individuals who frequently use cognitive reappraisal as an emotion regulation strategy may also be more prone to engage in emotional and stress-related eating behaviors. These findings suggest that cognitive reappraisal might be associated to emotional eating and stress eating behaviors, possibly reflecting a complex relationship where emotion regulation strategies are associated with eating responses to emotional and stress states. However, further research is needed to determine the underlying mechanisms of this association. The strong negative correlation between depression and the awareness subscale of the MEQ in the MB-EAT group suggests that individuals with greater depressive symptoms tend to have reduced mindful awareness while eating, consistent with previous studies (Yang et al., 2024). For instance, higher scores in the awareness of the MEQ have been associated with higher mental well-being (Khan & Zadeh, 2014). In the waitlist group, the only significant correlation found at post-test was a strong negative correlation between mindfulness and stress which aligns with the findings from previous studies (Hepburn et al., 2021) and indicates that increased mindfulness may serve as a protective factor against stress.

Mindfulness interventions teach individuals to pay attention, on purpose, in the present moment and to non-judgmentally be with their emotions. When one takes a mindful approach to difficult emotions, it provides an opportunity to change one’s habitual relationship to them and doing so frequently alleviates negative affect. Moreover, the levels of stress eating and mindful eating showed an inverse relationship with emotional responses, indicating a maladaptive pattern linked to stress eating. Notably, if individuals with emotional eating develop more mindful eating strategies during MB-EAT, they may transfer this maladaptive pattern into a more adaptive one (Kristeller et al., 2014). Individuals in the waitlist group, but not in the MB-EAT group, with more severe psychological problems exhibit less capability in mindful attention awareness. This discrepancy may indicate that separating a person's emotional state from their attention and awareness of their current experiences could help improve their emotional eating behaviors (Cavicchioli et al., 2021).

## Practical implications

The findings from this study show promising implications for clinical practice. First, the effectiveness of the MB-EAT intervention in reducing emotional eating underscores its potential as a tool for mental health professionals working with clients exhibiting emotional eating behaviors (Katterman et al., 2014). Second, the maladaptive eating pattern following emotional eating usually results in overweight/obesity, using MB-EAT as an advantageous precursor and/or adjunct to a weight-loss intervention for overweight/obesity. Therefore, using MB-EAT as a precursor and/or adjunct to a weight-loss intervention for populations with or without emotional eating would may lead to greater weight loss (Lattimore, 2020; Mason et al., 2016). Finally, incorporating mindfulness strategies into dietary practices could foster healthier relationships with food, which allows individuals to sustain long-term healthy eating behaviors.

## Limitations

The study is not without its limitations, which should be considered when interpreting the results. One notable limitation is the high attrition rate observed in the MB-EAT group, with only 56.5% of participants completing the 10-week program. The primary reasons for withdrawal included a lack of time to practice meditation and general preoccupation with studies, circumstances that are particularly relevant given that the participants were university students. This demographic often faces significant academic pressures, which likely contributed to their decision to discontinue the intervention. To enhance the feasibility of the program for this population, it may be beneficial to consider adapting the intervention to a shorter duration or a more flexible structure that accommodates their scheduling constraints. Another recommendation to improve adherence is to have more sessions of shorter duration or to include a few in-person sessions. Finally, the lack of extended follow-up is another limitation of the study. To assess the long-term effects of the intervention, follow-up assessments at 3 or 6 months could be incorporated.

Another limitation relates to the self-reported nature of meditation practice data. Due to considerable variability and inaccuracies in participants' reporting, exemplified by inconsistent recording, forgetting, or omitting practice times, this outcome was excluded from the analysis. This reliance on self-report introduces potential biases and affects the reliability of the data, highlighting the need for more objective measurement methods in future research. Future studies might use more objective methods of measuring meditation practice, such as using app-based tracking or physiological measures such as heart rate variability (Wei et al., 2025), to provide a more accurate assessment of participant engagement and the effect of mindfulness.

## Conclusions

MB-EAT is a helpful approach for university students who struggle with emotional eating. It can effectively help them manage their eating habits related to their emotions. The benefits of MB-EAT can grow over time, making it a lasting solution. As students practice mindfulness, they become better at handling their emotions and improve their overall mental health, which can help them overcome emotional eating.

## Data Availability

All relevant data are included within the manuscript and its supplementary materials. Additional datasets generated during the study are available from the corresponding author upon reasonable request.

## References

[CR1] Abdul Basir, S., Munirah, S. M., Abdul Manaf, Z., Ahmad, A., Mahadir, A., Abdul Kadir, N. B., Ismail, W. N. K., Mat Ludin, A. F., & Shahar, S. (2021). Reliability and validity of the Malay mindful eating questionnaire (MEQ-M) among overweight and obese adults. *International Journal of Environmental Research and Public Health,**18*(3), 1021. 10.3390/ijerph18031021 33498903 10.3390/ijerph18031021 PMC7908380

[CR2] Abdullah, A., Peeters, A., de Courten, M., & Stoelwinder, J. (2010). The magnitude of association between overweight and obesity and the risk of diabetes: A meta-analysis of prospective cohort studies. *Diabetes Research and Clinical Practice,**89*(3), 309–319. 10.1016/j.diabres.2010.04.012 20493574 10.1016/j.diabres.2010.04.012

[CR3] Baer, R. A., Smith, G. T., Hopkins, J., Krietemeyer, J., & Toney, L. (2006). Using self-report assessment methods to explore facets of mindfulness. *Assessment,**13*(1), 27–45. 10.1177/1073191105283504 16443717 10.1177/1073191105283504

[CR4] Benjamini, Y., & Hochberg, Y. (1995). Controlling the false discovery rate: A practical and powerful approach to multiple testing. *Journal of the Royal Statistical Society Series B: Statistical Methodology,**57*(1), 289–300. 10.1111/j.2517-6161.1995.tb02031.x

[CR5] Brown, K. W., & Ryan, R. M. (2003). The benefits of being present: Mindfulness and its role in psychological well-being. *Journal of Personality and Social Psychology,**84*(4), 822–848. 10.1037/0022-3514.84.4.822 12703651 10.1037/0022-3514.84.4.822

[CR6] Brown, K. W., Ryan, R. M., & Creswell, J. D. (2007). Mindfulness: Theoretical foundations and evidence for its salutary effects. *Psychological Inquiry,**18*(4), 211–237. 10.1080/10478400701598298

[CR7] Burnatowska, E., Wikarek, A., Oboza, P., Ogarek, N., Glinianowicz, M., Kocelak, P., & Olszanecka-Glinianowicz, M. (2023). Emotional eating and binge eating disorders and night eating syndrome in polycystic ovary syndrome—A vicious circle of disease: A systematic review. *Nutrients,**15*(2), 295. 10.3390/nu15020295 36678165 10.3390/nu15020295 PMC9865055

[CR8] Cao, C.-H., Liao, X.-L., Jiang, X.-Y., Li, X.-D., Chen, I.-H., & Lin, C.-Y. (2023). Psychometric evaluation of the depression, anxiety, and stress scale-21 (DASS-21) among Chinese primary and middle school teachers. *BMC Psychology,**11*(1), Article 209. 10.1186/s40359-023-01242-y 37452365 10.1186/s40359-023-01242-y PMC10349442

[CR9] Cardi, V., Leppanen, J., & Treasure, J. (2015). The effects of negative and positive mood induction on eating behaviour: A meta-analysis of laboratory studies in the healthy population and eating and weight disorders. *Neuroscience & Biobehavioral Reviews,**57*, 299–309. 10.1016/j.neubiorev.2015.08.011 26299807 10.1016/j.neubiorev.2015.08.011

[CR10] Cavicchioli, M., Scalabrini, A., Northoff, G., Mucci, C., Ogliari, A., & Maffei, C. (2021). Dissociation and emotion regulation strategies: A meta-analytic review. *Journal of Psychiatric Research,**143*, 370–387. 10.1016/j.jpsychires.2021.09.011 34592484 10.1016/j.jpsychires.2021.09.011

[CR11] Cheng, S.-H., & Wong, S. E. (2021). Stress, emotional eating and food choices among university students during the COVID-19 pandemic. *Malaysian Journal of Social Sciences and Humanities (MJSSH),**6*(9), Article Article 9. 10.47405/mjssh.v6i9.983

[CR12] Dakanalis, A., Mentzelou, M., Papadopoulou, S. K., Papandreou, D., Spanoudaki, M., Vasios, G. K., Pavlidou, E., Mantzorou, M., & Giaginis, C. (2023). The association of emotional eating with overweight/obesity, depression, anxiety/stress, and dietary patterns: A review of the current clinical evidence. *Nutrients,**15*(5), Article 1173. 10.3390/nu15051173 36904172 10.3390/nu15051173 PMC10005347

[CR13] DATATab team. (2025). *DATAtab: Online statistics calculator*. DATAtab e.U.

[CR14] Debeuf, T., Verbeken, S., Boelens, E., Volkaert, B., Van Malderen, E., Michels, N., & Braet, C. (2020). Emotion regulation training in the treatment of obesity in young adolescents: Protocol for a randomized controlled trial. *Trials,**21*(1), Article 153. 10.1186/s13063-019-4020-1 32039739 10.1186/s13063-019-4020-1 PMC7011608

[CR15] Du, C., Adjepong, M., Zan, M. C. H., Cho, M. J., Fenton, J. I., Hsiao, P. Y., Keaver, L., Lee, H., Ludy, M.-J., Shen, W., Swee, W. C. S., Thrivikraman, J., Amoah-Agyei, F., de Kanter, E., Wang, W., & Tucker, R. M. (2022). Gender differences in the relationships between perceived stress, eating behaviors, sleep, dietary risk, and body mass index. *Nutrients,**14*(5), Article 1045. 10.3390/nu14051045 35268020 10.3390/nu14051045 PMC8912409

[CR16] Duane, A., Casimir, A. E., Mims, L. C., Kaler-Jones, C., & Simmons, D. (2021). Beyond deep breathing: A new vision for equitable, culturally responsive, and trauma-informed mindfulness practice. *Middle School Journal,**52*(3), 4–14. 10.1080/00940771.2021.1893593

[CR17] Evers, C., Adriaanse, M., de Ridder, D. T. D., & de Witt Huberts, J. C. (2013). Good mood food. Positive emotion as a neglected trigger for food intake. *Appetite,**68*, 1–7. 10.1016/j.appet.2013.04.007 23602962 10.1016/j.appet.2013.04.007

[CR18] Fong, B. Y. F., Wong, M. C. S., Law, V. T. S., Lo, M. F., Ng, T. K. C., Yee, H. H. L., Leung, T. C. H., & Ho, P. W. T. (2020). Relationships between physical and social behavioural changes and the mental status of homebound residents in Hong Kong during the COVID-19 pandemic. *International Journal of Environmental Research and Public Health,**17*(18), Article Article 18. 10.3390/ijerph17186653 10.3390/ijerph17186653 PMC7559497 32932641

[CR19] Framson, C., Kristal, A. R., Schenk, J. M., Littman, A. J., Zeliadt, S., & Benitez, D. (2009). Development and validation of the mindful eating questionnaire. *Journal of the American Dietetic Association,**109*(8), 1439–1444. 10.1016/j.jada.2009.05.006 19631053 10.1016/j.jada.2009.05.006 PMC2734460

[CR20] Godet, A., Fortier, A., Bannier, E., Coquery, N., & Val-Laillet, D. (2022). Interactions between emotions and eating behaviors: Main issues, neuroimaging contributions, and innovative preventive or corrective strategies. *Reviews in Endocrine & Metabolic Disorders,**23*(4), 807–831. 10.1007/s11154-021-09700-x 34984602 10.1007/s11154-021-09700-x

[CR21] Grajek, M., Krupa-Kotara, K., Białek-Dratwa, A., Staśkiewicz, W., Rozmiarek, M., Misterska, E., & Sas-Nowosielski, K. (2022). Prevalence of emotional eating in groups of students with varied diets and physical activity in Poland. *Nutrients,**14*(16), Article 3289. 10.3390/nu14163289 36014794 10.3390/nu14163289 PMC9414995

[CR22] Gross, J. J., & John, O. P. (2003). Individual differences in two emotion regulation processes: Implications for affect, relationships, and well-being. *Journal of Personality and Social Psychology,**85*(2), 348–362. 10.1037/0022-3514.85.2.348 12916575 10.1037/0022-3514.85.2.348

[CR23] He, J., Chen, G., Wu, S., Niu, R., & Fan, X. (2020). Patterns of negative emotional eating among Chinese young adults: A latent class analysis. *Appetite,**155*, Article 104808. 10.1016/j.appet.2020.104808 32712196 10.1016/j.appet.2020.104808

[CR24] Henry, J. D., & Crawford, J. R. (2005). The short-form version of the depression anxiety stress scales (DASS-21): Construct validity and normative data in a large non-clinical sample. *The British Journal of Clinical Psychology,**44*(Pt 2), 227–239. 10.1348/014466505X29657 16004657 10.1348/014466505X29657

[CR25] Hepburn, S.-J., Carroll, A., & McCuaig, L. (2021). The relationship between mindful attention awareness, perceived stress, and subjective wellbeing. *International Journal of Environmental Research and Public Health,**18*(23), Article 12290. 10.3390/ijerph182312290 34886026 10.3390/ijerph182312290 PMC8656828

[CR26] Kabat-Zinn, J. (2003). Mindfulness-based interventions in context: Past, present, and future. *Clinical Psychology: Science and Practice,**10*, 144–156. 10.1093/clipsy.bpg016

[CR27] Katterman, S. N., Kleinman, B. M., Hood, M. M., Nackers, L. M., & Corsica, J. A. (2014). Mindfulness meditation as an intervention for binge eating, emotional eating, and weight loss: A systematic review. *Eating Behaviors,**15*(2), 197–204. 10.1016/j.eatbeh.2014.01.005 24854804 10.1016/j.eatbeh.2014.01.005

[CR28] Khan, Z., & Zadeh, Z. F. (2014). Mindful eating and its relationship with mental well-being. Procedia - Social and Behavioral Sciences, 5th World Conference on Psychology, Counseling and Guidance, WCPCG-2014, 1–3 May 2014, Dubrovnik, Croatia, 159, 69–73. 10.1016/j.sbspro.2014.12.330

[CR29] Kiken, L. G., Garland, E. L., Bluth, K., Palsson, O. S., & Gaylord, S. A. (2015). From a state to a trait: Trajectories of state mindfulness in meditation during intervention predict changes in trait mindfulness. *Personality and Individual Differences,**81*, 41–46. 10.1016/j.paid.2014.12.044 25914434 10.1016/j.paid.2014.12.044 PMC4404745

[CR30] Kristeller, J. L., & Wolever, R. Q. (2014). Chapter 6 - Mindfulness-based eating awareness training: Treatment of overeating and obesity. In R. A. Baer (Ed.), Mindfulness-based treatment approaches (2nd ed., pp. 119–139). Academic Press. 10.1016/B978-0-12-416031-6.00006-2

[CR31] Kristeller, J. L., & Hallett, C. B. (1999). An exploratory study of a meditation-based intervention for binge eating disorder. *Journal of Health Psychology,**4*(3), 357–363. 10.1177/135910539900400305 22021603 10.1177/135910539900400305

[CR32] Kristeller, J. L., & Jordan, K. D. (2018). Mindful eating: Connecting with the wise self, the spiritual self. *Frontiers in Psychology,**9*, Article 1271. 10.3389/fpsyg.2018.01271 30154740 10.3389/fpsyg.2018.01271 PMC6102380

[CR33] Kristeller, J., Wolever, R. Q., & Sheets, V. (2014). Mindfulness-based eating awareness training (MB-EAT) for binge eating: A randomized clinical trial. *Mindfulness,**5*(3), 282–297. 10.1007/s12671-012-0179-1

[CR34] Kwok, J. M. Y., & Ng, D. K. S. (2016). A study of the perceived stress level of university students in Hong Kong. *International Journal of Psychological Studies,**8*(4), Article Article 4. 10.5539/ijps.v8n4p91

[CR35] Larionow, P., Ocalewski, J., Mudło-Głagolska, K., Michalak, M., & Mazur, M. (2025). The clinical significance of cognitive reappraisal and expressive suppression across positive and negative emotions: Evidence on the Polish version of the emotion regulation questionnaire – Positive/Negative (ERQ-PN). *Frontiers in Psychiatry*. 10.3389/fpsyt.2025.1614234 40661887 10.3389/fpsyt.2025.1614234 PMC12257552

[CR36] Lattimore, P. (2020). Mindfulness-based emotional eating awareness training: Taking the emotional out of eating. *Eating and Weight Disorders - Studies on Anorexia, Bulimia and Obesity,**25*(3), 649–657. 10.1007/s40519-019-00667-y 10.1007/s40519-019-00667-y PMC7256094 30859465

[CR37] Lau, M. A., Bishop, S. R., Segal, Z. V., Buis, T., Anderson, N. D., Carlson, L., Shapiro, S., Carmody, J., Abbey, S., & Devins, G. (2006). The Toronto mindfulness scale: Development and validation. *Journal of Clinical Psychology,**62*(12), 1445–1467. 10.1002/jclp.20326 17019673 10.1002/jclp.20326

[CR38] Levin, M. E., Dalrymple, K., Himes, S., & Zimmerman, M. (2014). Which facets of mindfulness are related to problematic eating among patients seeking bariatric surgery? *Eating Behaviors,**15*(2), 298–305. 10.1016/j.eatbeh.2014.03.012 24854822 10.1016/j.eatbeh.2014.03.012

[CR39] Ling, J., & Zahry, N. R. (2021). Relationships among perceived stress, emotional eating, and dietary intake in college students: Eating self-regulation as a mediator. *Appetite,**163*, Article 105215. 10.1016/j.appet.2021.105215 33774134 10.1016/j.appet.2021.105215

[CR40] Liu, H., Yang, Q., Luo, J., Ouyang, Y., Sun, M., Xi, Y., Yong, C., Caihong, X., & Lin, Q. (2020). Association between emotional eating, depressive symptoms and laryngopharyngeal reflux symptoms in college students: A cross-sectional study in Hunan. *Nutrients,**12*(6), 1595. 10.3390/nu12061595 32485841 10.3390/nu12061595 PMC7352624

[CR41] Lovibond, S. H., & Lovibond, P. F. (1995). Manual for the depression anxiety stress scales. In Psychology Foundation of Australia (Vol. 56).

[CR42] Macht, M. (2022). Reduction of emotional eating with a mindfulness-based group training: A randomized controlled experience-sampling study. *Research Square*. 10.21203/rs.3.rs-1834873/v1

[CR43] Mason, A. E., Epel, E. S., Aschbacher, K., Lustig, R. H., Acree, M., Kristeller, J., Cohn, M., Dallman, M., Moran, P. J., Bacchetti, P., Laraia, B., Hecht, F. M., & Daubenmier, J. (2016). Reduced reward-driven eating accounts for the impact of a mindfulness-based diet and exercise intervention on weight loss: Data from the SHINE randomized controlled trial. *Appetite,**100*, 86–93. 10.1016/j.appet.2016.02.009 26867697 10.1016/j.appet.2016.02.009 PMC4799744

[CR44] Mercado, D. R., Lauren, G., Gemma, W., Jessica, W., Campbell, I. C., & Schmidt, U. (2021). The outcomes of mindfulness-based interventions for obesity and binge eating disorder: A meta-analysis of randomized controlled trials. *Appetite,**166*, Article 105464. 10.1016/j.appet.2021.105464 34146647 10.1016/j.appet.2021.105464

[CR45] Meule, A., Reichenberger, J., & Blechert, J. (2018a). Development and preliminary validation of the Salzburg emotional eating scale. *Frontiers in Psychology*. 10.3389/fpsyg.2018.00088 29467700 10.3389/fpsyg.2018.00088 PMC5807910

[CR46] Meule, A., Reichenberger, J., & Blechert, J. (2018b). Development and preliminary validation of the Salzburg stress eating scale. *Appetite,**120*, 442–448. 10.1016/j.appet.2017.10.003 28986162 10.1016/j.appet.2017.10.003

[CR47] Muharrani, N. P., Achmad, E. K., & Sudiarti, T. (2018). Effects of restrained, external, and emotional eating styles on weight gain among female students at Faculty of Public Health, Universitas Indonesia. *KnE Life Sciences,**4*(1), Article 8. 10.18502/kls.v4i1.1361

[CR48] Pedrelli, P., Nyer, M., Yeung, A., Zulauf, C., & Wilens, T. (2015). College students: Mental health problems and treatment considerations. *Academic Psychiatry,**39*(5), 503. 10.1007/S40596-014-0205-9 25142250 10.1007/s40596-014-0205-9 PMC4527955

[CR49] Portney, L. G., & Watkins, M. P. (2017). In Foundations of clinical research: Applications to practice (3rd ed.). McGraw-Hill Education. https://fadavispt.mhmedical.com/content.aspx?aid=1138249379

[CR50] Preece, D. A., Becerra, R., Robinson, K., & Gross, J. J. (2020). The emotion regulation questionnaire: Psychometric properties in general community samples. *Journal of Personality Assessment,**102*(3), 348–356. 10.1080/00223891.2018.1564319 30714818 10.1080/00223891.2018.1564319

[CR51] Riaz, H., Khan, M. S., Siddiqi, T. J., Usman, M. S., Shah, N., Goyal, A., Khan, S. S., Mookadam, F., Krasuski, R. A., & Ahmed, H. (2018). Association between obesity and cardiovascular outcomes: A systematic review and meta-analysis of Mendelian randomization studies. *JAMA Network Open,**1*(7), Article e183788. 10.1001/jamanetworkopen.2018.3788 30646365 10.1001/jamanetworkopen.2018.3788 PMC6324374

[CR52] Richardson, J. T. E. (2011). Eta squared and partial eta squared as measures of effect size in educational research. *Educational Research Review,**6*(2), 135–147. 10.1016/j.edurev.2010.12.001

[CR53] Salias, C., Gross, J. J., Petrova, K., Forbush, K. T., & Preece, D. A. (2025). Eating disorder symptoms and profiles of emotion regulation strategy use. *Journal of Affective Disorders,**380*, 94–103. 10.1016/j.jad.2025.03.050 40088985 10.1016/j.jad.2025.03.050

[CR54] Salvo, V., Curado, D. F., Sanudo, A., Kristeller, J., Schveitzer, M. C., Favarato, M. L., Isidoro, W., & Demarzo, M. (2022). Comparative effectiveness of mindfulness and mindful eating programmes among low-income overweight women in primary health care: A randomised controlled pragmatic study with psychological, biochemical, and anthropometric outcomes. *Appetite,**177*, Article 106131. 10.1016/j.appet.2022.106131 35753441 10.1016/j.appet.2022.106131

[CR55] Shek, D. T. L., Dou, D., & Zhu, X. (2022). Prevalence and correlates of mental health of university students in Hong Kong: What happened one year after the occurrence of COVID-19? *Frontiers in Public Health*. 10.3389/fpubh.2022.857147 35844893 10.3389/fpubh.2022.857147 PMC9277093

[CR56] Smith, J., Ang, X. Q., Giles, E. L., & Traviss-Turner, G. (2023). Emotional eating interventions for adults living with overweight or obesity: A systematic review and meta-analysis. *International Journal of Environmental Research and Public Health,**20*(3), Article Article 3. 10.3390/ijerph20032722 10.3390/ijerph20032722 PMC9915727 36768088

[CR57] Sze, K. Y. P., Lee, E. K. P., Chan, R. H. W., & Kim, J. H. (2021). Prevalence of negative emotional eating and its associated psychosocial factors among urban Chinese undergraduates in Hong Kong: A cross-sectional study. *BMC Public Health,**21*(1), Article 583. 10.1186/s12889-021-10531-3 33761930 10.1186/s12889-021-10531-3 PMC7988990

[CR58] Tang, Y.-Y., Hölzel, B. K., & Posner, M. I. (2016). Traits and states in mindfulness meditation. *Nature Reviews. Neuroscience,**17*(1), 1. 10.1038/nrn.2015.7 10.1038/nrn.2015.7 26631928

[CR59] Ungvari, Z., Fekete, M., Varga, P., Lehoczki, A., Fekete, J. T., Ungvari, A., & Győrffy, B. (2024). Overweight and obesity significantly increase colorectal cancer risk: A meta-analysis of 66 studies revealing a 25–57% elevation in risk. *GeroScience*. 10.1007/s11357-024-01375-x 39379738 10.1007/s11357-024-01375-x PMC12181496

[CR60] van Strien, T., Frijters, J. E. R., Bergers, G. P. A., & Defares, P. B. (1986). The Dutch eating behavior questionnaire (DEBQ) for assessment of restrained, emotional, and external eating behavior. *International Journal of Eating Disorders,**5*(2), 295–315. https://doi.org/10.1002/1098-108X(198602)5:2%3c295::AID-EAT2260050209%3e3.0.CO;2-T

[CR61] Warren, J. M., Smith, N., & Ashwell, M. (2017). A structured literature review on the role of mindfulness, mindful eating and intuitive eating in changing eating behaviours: Effectiveness and associated potential mechanisms. *Nutrition Research Reviews,**30*(2), 272–283. 10.1017/S0954422417000154 28718396 10.1017/S0954422417000154

[CR62] Watford, T. S., Braden, A. L., & Emley, E. A. (2019). Mediation of the association between mindfulness and emotional eating among overweight individuals. *Mindfulness,**10*(6), 1153–1162. 10.1007/s12671-018-1064-3

[CR63] Wei, Y., Xu, Y., Chen, W., Zheng, J., Chen, H., & Chen, S. (2025). Can heart rate variability demonstrate the effects and the levels of mindfulness? A repeated-measures study on experienced and novice mindfulness practitioners. *BMC Complementary Medicine and Therapies,**25*, 231. 10.1186/s12906-025-04972-1 40611081 10.1186/s12906-025-04972-1 PMC12225205

[CR64] Xu, Y., Song, J., Ren, Y., Barnhart, W. R., Dixit, U., Ji, F., Chen, C., & He, J. (2024). Negative emotional eating patterns in general Chinese adults: A replication and expansion study examining group differences in eating disorder symptomatology, psychosocial impairment, and emotion regulation difficulties. *Eating Behaviors,**54*, Article 101899. 10.1016/j.eatbeh.2024.101899 38936286 10.1016/j.eatbeh.2024.101899

[CR65] Yang, M., Wang, X., Zhang, Y., Qian, W., & Tang, Y. (2024). Mindfulness acting with awareness and emotional eating among polycystic ovary syndrome women with infertility: The mediating role of depression. *Frontiers in Psychology*. 10.3389/fpsyg.2024.1499705 39723408 10.3389/fpsyg.2024.1499705 PMC11669249

[CR66] Zeidan, F., Baumgartner, J. N., & Coghill, R. C. (2019). The neural mechanisms of mindfulness-based pain relief: A functional magnetic resonance imaging-based review and primer. *Pain Reports,**4*(4), Article e759. 10.1097/PR9.0000000000000759 31579851 10.1097/PR9.0000000000000759 PMC6728003

[CR67] Zhou, L., Zhang, N., Zhou, Y., & Xu, W. (2020). The dynamic relationship between state mindfulness and negative emotions. *PsyCh Journal,**9*(6), 903–910. 10.1002/pchj.385 32909384 10.1002/pchj.385

